# Giant Baker's Cyst Associated with Rheumatoid Arthritis

**DOI:** 10.1155/2017/4293104

**Published:** 2017-01-02

**Authors:** Levent Adiyeke, Emre Bılgın, Tahir Mutlu Duymus, İsmail Emre Ketencı, Meriç Ugurlar

**Affiliations:** ^1^Haydarpasa Numune Training and Research Hospital, Department of Orthopaedics and Traumatology, İstanbul, Turkey; ^2^Tepecik Education and Research Hospital, Department of Orthopaedics and Traumatology, Izmir, Turkey; ^3^Şişli Etfal Training and Research Hospital, Department of Orthopaedics and Traumatology, İstanbul, Turkey

## Abstract

We report a rare case of a “giant Baker's cyst-related rheumatoid arthritis (RA)” with 95 × 26 mm dimensions originating from the semimembranosus tendon. The patient presented with chronic pain and a palpable mass behind his left calf located between the posteriosuperior aspect of the popliteal fossa and the distal third of the calf. In MRI cystic lesion which was located in soft tissue at the posterior of gastrocnemius, extensive synovial pannus inside and degeneration of medial meniscus posterior horn were observed. Arthroscopic joint debridement and partial excision of the cyst via biomechanical valve excision were performed. The patient continued his follow-up visits at Rheumatology Department and there was no recurrence of cyst-related symptoms in 1-year follow-up. Similar cases were reported in the literature previously. However, as far as we know, a giant Baker's cyst-related RA, which was treated as described, has not yet been presented.

## 1. Introduction

Rheumatoid arthritis (RA) is a common chronic inflammatory autoimmune disease that affects 3% of females and 1% of males. This condition is characterized by neutrophil infiltration of soft tissues and hypertrophy of the joint capsule, synovia, tendon sheath, and bursa due to chronic inflammation [1]. Villi formation and intra-articular pannus formation develop as the amount of synovial fluid increases, leading to the proliferation of synovial tissue. Knee joints are the most commonly affected joints in 15% of RA patients [2, 3]. The joint capsule becomes tightened, which is associated with increasing amounts of synovial fluid. A palpable mass behind the knee joint, which typically expands the subcutaneous tissue, occurs between the semimembranosus and medial head of the gastrocnemius tendons [4, 5]. In this report, we present a giant Baker's cyst in a RA patient expanding to the middle of the calf.

## 2. Case

A 42-year-old male with left knee pain lasting for 9 months and swelling behind the knee was admitted to the orthopedics clinic. He stated that he had increased pain with standing and limited knee flexion. He had been receiving IV methotrexate (Metoject 20 mg/2 mL 1 × 1 per week) and oral sulfasalazine (Salazopyrin-en 500 Mg 2 × 2 per day) for 3 months for the RA. During physical examination, a large mass was observed behind the left knee, expanding to the proximal calf. The mass was immobile and soft when palpated. The range of motion (ROM) of the left knee was Fl/Ext 120/−10 degree. McMurray and Patellar Ballottement tests were positive. Neurovascular examination of the left lower extremity was normal. Laboratory studies showed the following: ESR, 18 mm/h; CRP, 5 mm/dL; and WBC, 10.000.

**Figure 1:**
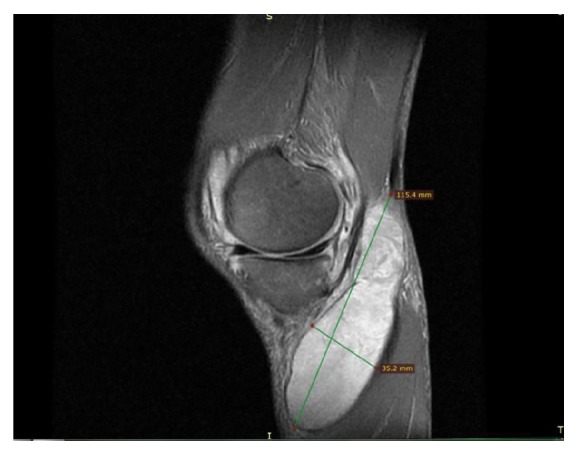
Magnetic resonance imaging sagittal plane view: cyst size.

**Figure 2:**
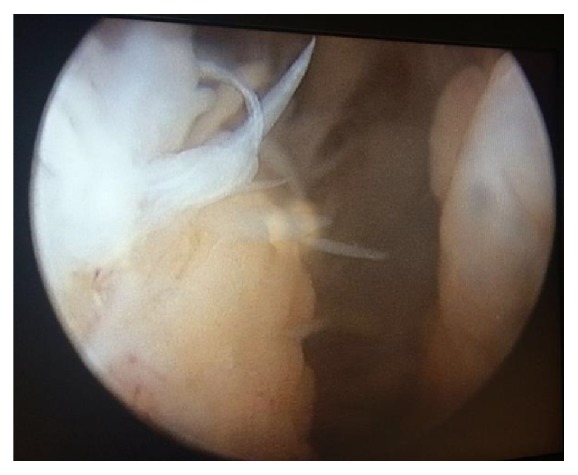
Arthroscopic view of pannus formation.

**Figure 3:**
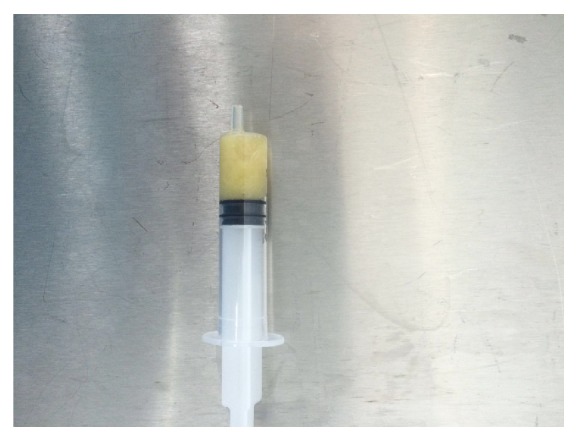
Cyst aspiration material compatible with subacute inflammatory period.

**Figure 4:**
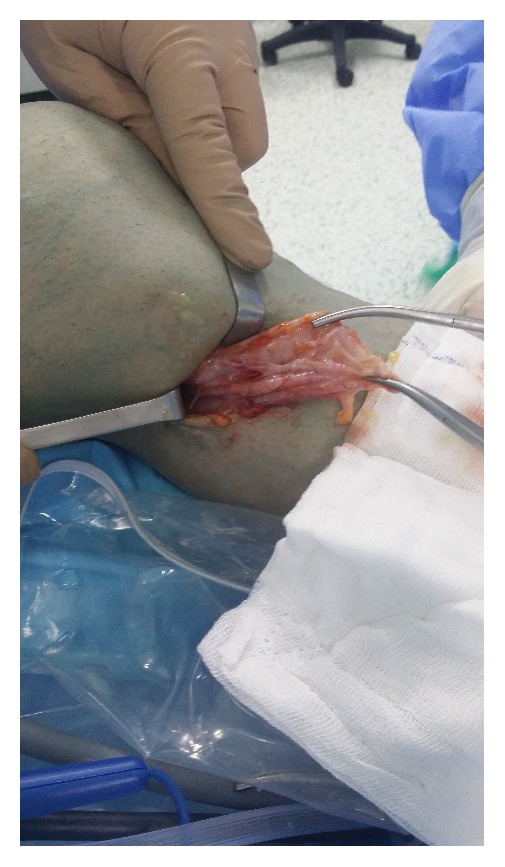
Excision of the Baker's cyst by posteromedial incision.

Minimal degenerative changes were observed at the medial plateau based on plain radiographs. Based on magnetic resonance imaging (MRI), a 95 mm × 26 mm cystic lesion had located in soft tissue at the posterior of the gastrocnemius, with extensive synovial pannus inside; degeneration of the medial meniscus posterior horn was observed (Figure 1). Diagnostic arthroscopy was performed and intense synovial hypertrophy signs and pannus formation were detected (Figure 2). Samples were obtained from synovial tissue for biopsy. Low-viscosity cyst material flowed into the joint after controlling for the horizontal tear of the medial meniscus posterior horn with a probe (Figure 3). The canal from which the cyst content originated was expanded with a clamp, and the canal stoma was debrided using a shaver-blade. The cyst was reached by creating an approximately 9 cm posteromedial incision (Figure 4). The cyst was released from surrounding soft tissues, excised, and sent to pathology. Histopathological examination showed fibrohyalinized tissue covered with synovial epithelium. We also observed plasma cells due to active chronic inflammation, as well as fibrovascular tissue fragments compatible with the cyst wall. Analysis of synovial fluid showed the following: WBC, 27200; PMN (%), 75; glucose, 67; and culture negative. Pain and swelling decreased after discharge, and full ROM of the left knee was observed at follow-up visits.

## 3. Discussion

RA is a chronic immune system-induced disease that leads to increased production of synovial fluid depending on inflammation in the knee joint. In this disease in which synovial tissue has a strong effect on the knee joint, chondral damage and connective tissue involvement may be present [1, 6, 7]. This disease is associated with increased synovial fluid in the knee and can lead to the formation of a Baker's cyst, with a one-way valve mechanism formed in the knee joint. Baker's cyst, which is generally palpated with asymptomatic swelling in the popliteal fossa, may occur depending on meniscal tears and arthrosis [8, 9]. In a study by Fielding et al., which was performed with MRI, they stated that Baker's cyst was seen in adult populations at a rate of 4%; this rate was higher in the elderly population [10] and is even higher in diseases such as RA and gout. In situations involving pain, swelling in the popliteal fossa and severe limitation of knee ROM, further evaluation is required. Deep vein thrombosis, popliteal artery aneurysm and cyst rupture should be considered in the differential diagnosis [9, 11–14]. The differential diagnosis should also include malignant tumors, which can settle in the popliteal region and have cystic characteristics (synovial sarcoma, fibrosarcoma, and malignant fibrous histiocytoma). To evaluate Baker's cyst, MRI and ultrasound (US) are the most important diagnostic tools in terms of the exclusion of different diagnoses and characterizing the relationship of cyst content with the joint [8, 10, 15, 16].

For the treatment of Baker's cyst, various conservative and surgical treatments were applied depending on the underlying cause and accompanying pathology. Within the conservative applications, successful results were reported with joint aspiration and corticosteroid (KS) injection in osteoarthritis related cases [6, 17–19]. Acebes et al. reported successful results following aspiration of cyst contents and KS injection in osteoarthritis related cases [17]. In similar cases, Bandinelli et al. also reported successful results using US-guided steroid injection directly into the cyst and intraarticular steroid injection; both methods were considered effective [18]. In the treatment of Baker's cyst accompanied with RA, Hofman-González et al. stated that methotrexate application to the cyst may be an alternative method for patients with surgical risk factors [20]. In our case, active complaints of the patient were present, despite ongoing IV methotrexate treatment.

Although successful results were obtained in Baker's cyst treatment with conservative methods, surgical interventions may be required. Cyst excision is one of the options for surgical interventions, although extensive exposure is required. However, arthroscopic methods have become more popular. By performing arthroscopy, intra-articular pathology leading to cyst formation can be intervened, and biomechanical valve mechanisms thought to be responsible for cyst formation can be treated [21, 22]. Rupp et al. indicated that Baker's cyst is often accompanied by a medial meniscal tear and chondral lesions. It was reported that regression of the cyst was observed after performing arthroscopic debridement, partial meniscectomy, and microfracture for low-grade chondral lesions. However, formation of an effusion was not adequately controlled and treatment success was limited in grades 3 and 4 chondral lesions [23].

In a previous study, Sansone and De Ponti obtained good results following arthroscopic treatment of biomechanical valves and accompanying intra-articular disorders in a series of 30 patients with a two-year follow-up. Moreover, 95% of patients benefited from this method [22]. In a similar study, Calvisi et al. stated that, after suturing the canal valve using the all-inside arthroscopic technique with anterolateral and posteromedial portals and treatment of the intra-articular conditions, Baker's cyst disappeared in 64% of cases after two-year follow-up. Regression of the cyst was observed at a rate of 27%, and the cyst progressed in 9% of cases [24].

Intervention of intra-articular disorders and expansion of canal diameter with excision of the canal valve by performing arthroscopy is a recently accepted method. Ahn et al. stated that they achieved 94% success in a three-year follow-up period using this method [25]. In a study by Cho, using a similar method, good clinical results were obtained in a two-year follow-up with no recurrence [26]. In our case, the canal diameter of the cyst was expanded and intra-articular pathology was treated by shaving the medial meniscus posterior horn through arthroscopy. Symptoms related to the Baker's cyst, which extended to the posteromedial calf, were eliminated by partial cyst excision. The patient continued their follow-up visits at the Rheumatology Department, and there was no recurrence of cyst-related symptoms during a 1-year follow-up.

Successful results can be obtained by performing arthroscopic debridement and biomechanical valve excision in cases with Baker's cyst accompanied by intra-articular damage. Additionally, open cyst excision should be considered in symptomatic and recurrent cases.

## References

[1] Ozsoy M. H., Altınel L., Basarır K., Cavuoğlu A., Dincel V. (2006). Romatoid artritte eklem hastalığının patogenezi. *TOTBID Dergisi*.

[2] Chalmers P. N., Sherman S. L., Raphael B. S., Su E. P. (2011). Rheumatoid synovectomy: does the surgical approach matter?. *Clinical Orthopaedics and Related Research*.

[3] Triolo P., Rossi R., Rosso F., Blonna D., Castoldi F., Bonasia D. E. (2016). Arthroscopic synovectomy of the knee in rheumatoid arthritis defined by the 2010 ACR/EULAR criteria. *The Knee*.

[4] Fritschy D., Fasel J., Imbert J.-C., Bianchi S., Verdonk R., Wirth C. J. (2006). The popliteal cyst. *Knee Surgery, Sports Traumatology, Arthroscopy*.

[5] Martí-Bonmatí L., Mollá E., Dosdá R., Casillas C., Ferrer P. (2000). MR imaging of Baker cysts—prevalence and relation to internal derangements of the knee. *Magnetic Resonance Materials in Physics, Biology and Medicine*.

[6] Köroğlu M., Callıoğlu M., Eriş H. N. (2012). Ultrasound guided percutaneous treatment and follow-up of Baker's cyst in knee osteoarthritis. *European Journal of Radiology*.

[7] Nogueira E., Gomes A., Preto A., Cavaco-Paulo A. (2016). Update on therapeutic approaches for rheumatoid arthritis. *Current Medicinal Chemistry*.

[8] Liao S.-T., Chiou C.-S., Chang C.-C. (2010). Pathology associated to the Baker's cysts: A Musculoskeletal Ultrasound Study. *Clinical Rheumatology*.

[9] Artul S., Jabaly-Habib H., Artoul F., Habib G. (2015). The association between Baker's cyst and medial meniscal tear in patients with symptomatic knee using ultrasonography. *Clinical Imaging*.

[10] Fielding J. R., Franklin P. D., Kustan J. (1991). Popliteal cysts: a reassessment using magnetic resonance imaging. *Skeletal Radiology*.

[11] DeLuca P. F., Bartolozzi A. R. (1999). Tibial neuroma presenting as a Baker cyst. A case report. *The Journal of Bone & Joint Surgery—American Volume*.

[12] Gombert A., Jalaie H., Jacobs M. J., Grommes J. (2015). Popliteal artery aneurysma as an important differential diagnosis. *Sportverletzung-Sportschaden*.

[13] Kim J. S., Lim S. H., Hong B. Y., Park S. Y. (2014). Ruptured popliteal cyst diagnosed by ultrasound before evaluation for deep vein thrombosis. *Annals of Rehabilitation Medicine*.

[14] Alonso-Gómez N., Pérez-Piqueras A., Martínez-Izquierdo A., Sáinz-González F. (2015). Giant baker' cyst. Differential diagnosis of deep vein thrombosis. *Reumatologia Clinica*.

[15] Riente L., Delle Sedie A., Filippucci E. (2010). Ultrasound imaging for the rheumatologist XXVII. Sonographic assessment of the knee in patients with rheumatoid arthritis. *Clinical and Experimental Rheumatology*.

[16] De de Beer V. J., Bogoch E. R., Smythe H. A. (1990). Lymphoma presenting as a popliteal mass in a patient with rheumatoid arthritis. *Journal of Rheumatology*.

[17] Acebes J. C., Sánchez-Pernaute O., Díaz-Oca A., Herrero-Beaumont G. (2006). Ultrasonographic assessment of Baker's cysts after intra-articular corticosteroid injection in knee osteoarthritis. *Journal of Clinical Ultrasound*.

[18] Bandinelli F., Fedi R., Generini S. (2012). Longitudinal ultrasound and clinical follow-up of Baker's cysts injection with steroids in knee osteoarthritis. *Clinical Rheumatology*.

[19] Smith M. K., Lesniak B., Baraga M. G., Kaplan L., Jose J. (2015). Treatment of popliteal (baker) cysts with ultrasound-guided aspiration, fenestration, and injection: long-term follow-up. *Sports Health*.

[20] Hofman-González F., Hernández-Díaz C., Solano-Ávila C., López-Reyes A. G., Peña-Ayala A., Pineda-Villaseñor C. (2013). Giant Baker's cyst treated with intralesional methotrexate. *Cirugia y Cirujanos*.

[21] Pankaj A., Chahar D., Pathrot D. (2016). Arthroscopic management of popliteal cysts. *Indian Journal of Orthopaedics*.

[22] Sansone V., De Ponti A. (1999). Arthroscopic treatment of popliteal cyst and associated intra-articular knee disorders in adults. *Arthroscopy*.

[23] Rupp S., Seil R., Jochum P., Kohn D. (2002). Popliteal cysts in adults: prevalence, associated intraarticular lesions, and results after arthroscopic treatment. *American Journal of Sports Medicine*.

[24] Calvisi V., Lupparelli S., Giuliani P. (2007). Arthroscopic all-inside suture of symptomatic Baker's cysts: a technical option for surgical treatment in adults. *Knee Surgery, Sports Traumatology, Arthroscopy*.

[25] Ahn J. H., Lee S. H., Yoo J. C., Chang M. J., Park Y. S. (2010). Arthroscopic treatment of popliteal cysts: clinical and magnetic resonance imaging results. *Arthroscopy - Journal of Arthroscopic and Related Surgery*.

[26] Cho J. H. (2012). Clinical results of direct arthroscopic excision of popliteal cyst using a posteromedial portal. *Knee Surgery & Related Research*.

